# Patients Satisfied with Care Report Better Quality of Life and Self-Rated Health—Cross-Sectional Findings Based on Hospital Quality Data

**DOI:** 10.3390/healthcare11050775

**Published:** 2023-03-06

**Authors:** Linda Baumbach, Marc Frese, Martin Härter, Hans-Helmut König, André Hajek

**Affiliations:** 1 Department of Health Economics and Health Services Research, University Medical Center Hamburg-Eppendorf, 20246 Hamburg, Germany; 2 Office for Quality Management and Clinical Process Management, University Medical Centre Hamburg-Eppendorf, 20246 Hamburg, Germany; 3 Department of Medical Psychology, University Medical Center Hamburg-Eppendorf, 20246 Hamburg, Germany

**Keywords:** health status, inpatient care, quality of life, satisfaction with care, self-rated health, subjective health

## Abstract

Background: Satisfaction with care is an important indicator of health care quality. However, if this process measure is associated with patients’ outcomes in real-world data is largely unknown. We, therefore, aimed to evaluate if satisfaction with physician- and nurse-related care is associated with quality of life and self-rated health among inpatients at the University Hospital Hamburg-Eppendorf in Germany. Method: We used standard hospital quality survey data of 4925 patients treated at various departments. We used multiple linear regressions to examine an association between satisfaction with staff-related care and quality of life as well as self-rated health, adjusted for age, gender, mother tongue, and treating ward. Patients rated their satisfaction with physician- and nurse-related care from 0 “not at all” to 9 “very much”. The outcomes regarding quality of life and self-rated health were evaluated on five-point Likert scales ranking from 1 “bad” to 5 “excellent”. Results: We found that satisfaction with physician-related care was positively associated with quality of life (ß = 0.16; *p* < 0.001) as well as with self-rated health (ß = 0.16; *p* < 0.001). Similar findings were observed for satisfaction with nurse-related care and the two outcomes (ß = 0.13; *p* < 0.001 and ß = 0.14; *p* < 0.001, respectively). Conclusion: We show that patients who are more satisfied with staff-related care report better quality of life and self-rated health than patients less satisfied with care. Thus, patient satisfaction with care, is not only a process measure indicating the quality of care but is also positively associated with patient-reported outcomes.

## 1. Introduction

Patient-centered care is becoming more and more important in clinical practice. Hence, nowadays, health outcomes include the patient’s perspective, which is often obtained via patient-reported outcome measures (PROMS). Quality of life and self-rated health are examples of such measures. The former is defined as “… an individual’s perception of their position in life in the context of the culture and value systems in which they live and in relation to their goals, expectations, standards, and concerns” [1]. It involves, amongst others, physical well-being, material well-being, social well-being, emotional well-being, and development and activity [2]. Quality of life can predict changes in several diseases [3,4]. Self-rated health is another self-reported outcome that can predict mortality [5]. Consequently, quality of life and self-rated health are increasingly recognized as important and effective clinical PROMS.

Satisfaction with care is a patient-reported experience measure informing on the perceived received care from the patient’s point of view. It mirrors one part of the environment in which patients act. According to the international classification of functioning (ICF), which aims at describing an individual’s functioning and disabilities, such environmental factors act as barriers or facilitators to the person’s functioning [6]. Inpatients are in an exceptional situation in their life where they depend on the help of professionals. This dependency, as with any environmental factor, is expected to influence the patients’ ability to function. Key drivers of patients’ satisfaction relate to health professionals. Their connection and communication with patients are crucial [7]. Dissatisfied patients do not adhere to treatments and change their health professionals more often than satisfied patients [8]. Satisfaction with staff-related care might, therefore, be an indicator of health outcomes.

Several studies examined the association between satisfaction with care and quality of life [9,10,11]. In psychotic patients, these two measures are consistently positively associated [12]. Furthermore, patients undergoing hip and knee arthroplasty who are satisfied with their care report better recovery [13] and, in patients with stroke, satisfaction with care is an indicator of the patients’ quality of life [10]. Additionally, a longitudinal study involving dermatological patients suggests that patients’ satisfaction affects their subsequent quality of life [14]. However, these studies are all limited to illness-specific patient groups and mostly include only a few hundred patients. Thus, it remains unknown if satisfaction with care is generally associated with quality of life and self-rated health, or if this is only true for specific patient groups.

Hospitals have started to evaluate patients’ satisfaction with care on a daily basis. Medical service quality management sees patient satisfaction as a critical factor, since it mirrors the patients’ view on the quality of the provided service [15]. It helps identify areas in need of interventions and investments to improve the care’s quality. Satisfaction with care collected with characteristics and outcomes of the patients provides a unique opportunity to verify the idea of an overall association between satisfaction with care and the quality of life as well as self-rated health in inpatients.

In the present study, we aimed to investigate the association between inpatients’ satisfaction with physician- and nurse-related care and quality of life as well as self-rated health. We hypothesized that patients who are satisfied with their received care report a higher quality of life and better self-rated health in comparison to patients who are less satisfied with their received care.

## 2. Methods

### 2.1. Data Source

We used data from the continuous patient survey (KoPa) at the University Medical Center Hamburg-Eppendorf (UKE). The UKE is one of 36 university hospitals in Germany and treats about 90,000 inpatients annually. The KoPa was implemented in 2012 to measure and identify areas in need of interventions to secure and improve the quality of care. Posters and staff members inform and motivate inpatients to voluntarily participate in the KoPa close to their discharge. Patients respond to the survey via a tablet computer, which is attached to their hospital bed. Prior to responding to the questions, they consent to the usage of their data by the quality management of the hospital. However, the data collected do not contain any personal data and are consequently anonymous. Patients treated in the psychiatric, intensive, and child wards are not asked to participate in the KoPa, since their feedback is likely to be given by proxies. In 2020, major methodological changes were introduced in the KoPa. Now, it contains 49 items describing the patient characteristics as well as the received treatment and care at the hospital. In this project, we only use data collected after and throughout 2021.

Of the patients participating in the KoPa in 2021, we excluded patients who denied answering one or more questions.

### 2.2. Variables

The dependent variables of interest are quality of life and self-rated health. Both items stem from the PROMIS 1.1 Global Health and are obtained with a Likert scale [16]. Patients were asked “How would you describe your overall quality of life?” and “How would you describe your overall state of health?”. They could respond to each of the questions: “bad”, “fair”, “good”, “very good”, or “excellent”.

Our independent variables of interest measure the overall satisfaction with the received care from the physicians and the nurses. Participants responded to (a) “I am satisfied with the treatment and care received by the physicians” and (b) “I am satisfied with the treatment and care received by the nursing staff”—both on a scale from 0 “not at all” to 9 “very much”.

To identify confounding variables, we created a directed acyclic graph (Supplementary Figure S1). Considering this graph and respecting the availability of data, we included four covariates in our regression analyses: age, sex, mother tongue, and medical specialty/ward. The first three variables refer to the patients, whereas the last item refers to the characteristics of the hospital. Age was measured in the following categories “≤20 years”, “21–40 years”, “41–60 years”, “61–80 years”, “>80 years”. The sex could be reported as female, male, or diverse. Mother tongue was indicated as either German or other. Furthermore, for sex and mother tongue, patients could also deny their reply. Information regarding the treating ward was extracted from the questionnaire system. Wards were grouped into nine medical specialties (1) eyes, otorhinolaryngology, and maxillofacial or oral surgery, (2) internal medicine, (3) gynecology and obstetrics, (4) urology, including urological oncology, (5) oncology, (6) cardiology, heart, and vascular surgery, (7) neurology und neurosurgery, (8) general and trauma surgery, (9) dermatology, and (10) others. Patients treated at wards focusing on several specialties were grouped into “others”.

### 2.3. Statistical Methods

Descriptive statistics are presented as number and percentages for categorical variables and as mean and standard deviation for continuous variables. In the case of skewed distributions, we additionally report the median and interquartile range.

We performed adjusted multiple linear regressions to evaluate associations between the overall satisfaction with physician- and nurse-related care and quality of life as well as self-rated health. We investigated the collinearity among the explanatory variables with the variance inflation factor and found only values below 2. We observed no clear outliers in diagnostics plots. All statistical tests were performed at the 0.05 level of significance using R Studio Version 2022.12.0+353.

## 3. Results

### 3.1. Participants

In 2021, the response rate to the KoPa was about ten percent. This led to 6133 patients, who were treated at the included wards, finishing the survey. However, of these patients, we excluded 19.7% due to denying and missing information. Thus, our final sample consisted of 4925 patients. Their characteristics are presented in Table 1. We observed skewed distributions in satisfaction with physician- and nurse-related care. Quality of life and self-rated health were normally distributed and had means of 3.21 (SD: 0.90) and 3.01 (SD: 0.87), respectively. The number of participants from the wards varied between 80 and 1717. The difference in samples is due to different sizes of the wards but also due to varying average numbers of treated patients and promotion strategies at the wards.

### 3.2. Analyses

**Figure 1:**
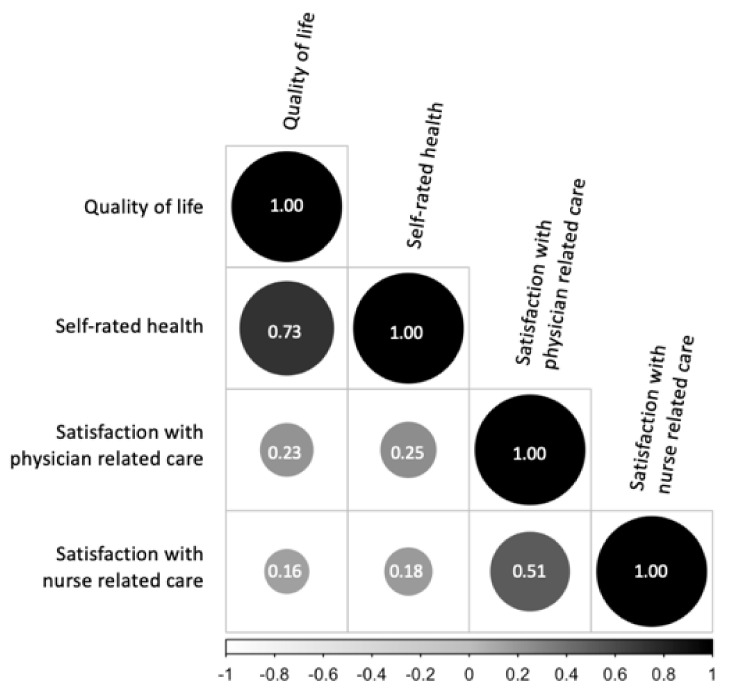
Visualization of the Spearman correlation coefficients of our variables of interest.

Following Akoglu’s definition, we found a fair correlation between satisfaction with physician- and nurse-related care (Figure 1) [17]. Our dependent variables were moderately correlated (r = 0.73). Consequently, we performed four adjusted multiple linear regressions, of which the most important information is summarized in Table 2. Here, we corrected our standard errors due to heteroscedasticity and thus calculated robust standard errors.

In regression analysis, we found that satisfaction with physician-related care was positively associated with quality of life (ß = 0.16; *p* < 0.001) and with self-rated health (ß = 0.16; *p* < 0.001). For satisfaction with nurse-related care, we found a positive association of ß = 0.13; *p* < 0.001 and ß = 0.14; *p* < 0.001, respectively. The complete results of our four regressions are provided in Supplementary material (Tables S1–S4).

## 4. Discussion

Based on a large sample of inpatients, our aim was to investigate the association between satisfaction with staff-related care and quality of life as well as self-rated health, independent of underlining health conditions. We observed high satisfaction rates for physician- as well as nurse-related care. Furthermore, we found that more satisfied patients report greater quality of life and self-rated health at the end of their hospital stay compared to patients less satisfied with staff-related care. Our study extends our current knowledge regarding these associations solely based on small, illness-specific samples.

For the interpretation of our findings, it is important to remember that our response rate was only about 10% and that we excluded another 20% of the sample due to missing information. The low response rate is, however, common for surveys like ours. Nonetheless, it leaves some uncertainties regarding generalizability. Furthermore, the exclusion of 20% may be criticized, however, this, again, is common in studies like the one presented.

Maybe due to these similarities in the sample and despite differences in study designs, our findings are in line with existing literature [9,10,13,14,15,18,19]. High satisfaction rates were also found in other studies based on data from medical service quality management [15,19]. This could suggest that patients are, in general, very satisfied with received care. However, external factors could also explain this observation, for example, the response bias. An investigation of differences in satisfaction between responders and non-responders revealed a J-shape curve [19]. Thus, very satisfied patients reply mostly to hospital surveys, while the least satisfied respond more often than the middle but still less often than the very satisfied patients [19]. However, at university hospitals, such associations were not found [19]. Another influencing external factor is the hospital size [15]. Patients from larger hospitals are less satisfied than patients from smaller hospitals [15]. However, neither of these two explanations holds true for the present study and thus explains our high satisfaction rates. An explanation for the high satisfaction rates with nurse-related care could be that patients treated at hospitals focusing on nursing care report higher satisfaction with nurse-related care [15]. Since our study took place at a hospital currently putting a lot of effort into nurse-related care, it might explain the high satisfaction rate.

Patient-related factors could also explain our high satisfaction rates [20]. Amongst others, marital status, previous admission, later response, and if a relative responded for the patient are factors associated with staff-related satisfaction [20]. Our data do not cover information on the two former variables. Thus, we can only speculate on their association with the selected outcomes. The term late response indicates if patients replied directly or after one or more reminders to the survey. Late responders are often less satisfied [20]. Unfortunately, we do not know for our sample if staff reminded patients to participate or not. Furthermore, the inpatients in our sample were recruited while they were still treated at the hospital, thus they might have indicated greater satisfaction rates to satisfy their treating staff. This special situation might also explain why our sample contained more male than female participants, which is uncommon in survey evaluations like this. Finally, if another person responds for a patient, lower satisfaction scores are given [20]. Since our sample did not include patients for which a proxy was likely to provide the answers, it partly explains our high satisfaction rates. Age and gender are further factors associated with patients’ satisfaction with care. Age is mostly positively correlated with satisfaction, and men are more often satisfied with received care than women. However, some studies do not confirm these observations [21,22,23]. Nonetheless, since our sample contains more men, this might partly explain our high satisfaction rates. Therefore, we adjusted our analyses for these variables.

An association between satisfaction with staff-related care and health outcomes has been investigated previously. These former investigations used research-based data but not data from medical service quality management [10,11,14,18]. They focused on specific patient groups while we investigated an overall association between staff-related satisfaction and quality of life and self-rated health in service quality management data. After adjusting for different medical wards, we still observed an association between our variables. Thus, more satisfied patients, independent of the treating ward, report better quality of life and self-rated health.

Positive associations between satisfaction and health outcomes were also observed in a literature review [24]. Though this review focused on the experience of patients, of which satisfaction with staff-related care is just one part, the involved studies on satisfaction indicate a positive association with health outcomes. Another study contradicts our findings regarding an association [25]. However, that study treated satisfaction with staff-related care as binary, either satisfied or dissatisfied, which reduces the information. Furthermore, the study sample consisted of a special patient group, those undergoing elective spine surgery, and finally, they performed phone interviews to obtain the data. Thus, there are several design-related factors that could explain the different findings.

In summary, the patient–staff interaction shapes part of the experienced environment in real-world clinical settings. If patients find the interactions with health staff more satisfying, they report better health outcomes. This corresponds with the idea of the ICF, suggesting that environmental factors influence the functioning and thus, patients’ health.

### Methodological Considerations

Similarities, as well as differences between our and similar studies, can be found in the utilized variables. Satisfaction with care is often, as in our study, evaluated on a Likert scale [13,18]. However, we used the overall satisfaction with physician- and nurse-related care, while other studies distinguish between specific aspects of satisfaction with care [11]. These detailed variables correlate with overall satisfaction with care. They are important to evaluate services and need to be respected in planning interventions to improve care. However, we aimed to establish knowledge on the overall relationship between the process measure satisfaction with staff-related care and health-related outcomes across several inpatient groups, which does not require information on specific aspects of satisfaction with care. Future in-depth studies may be conducted to understand the impact of satisfaction with medical care on quality of life and self-rated health to expand our knowledge further.

As in our evaluation, quality of life and self-rated health are commonly applied PROMS in medical studies. However, the chosen tools for our data do not account for specific dimensions of the constructs quality of life or self-rated health as other measures, such as the EQ-5D do [26]. For example, the EQ-5D only focuses on health-related quality of life aspects. The one-question items of our study were chosen to reduce the patients’ burden in responding. However, it is a limitation to our study. Consequently, we may only conclude that there is an overall association between satisfaction with staff-related care and quality of life as well as self-rated health.

In summary, the advantage of our utilized measures is the practicability that ongoing survey evaluations require. However, obtaining a deeper understanding of the aspects which influence satisfaction with care or which aspects of the quality of life and self-rated health need attention requires more detailed measures.

Another methodological consideration relates to our sample. We obtained data from one large University Medical Center in Germany, which limits the generalizability. Furthermore, the response rate was very low in our study. Thus, the possibility of a sample selection bias cannot be dismissed. Due to a lack of information on differences between patients who voluntarily participate in the survey compared to those who do not, our results can only be generalized to the former group. However, since we included patients from a variety of medical specialties, our findings hold true for several inpatient groups.

Finally, we only used cross-sectional data, which makes it difficult to clarify the causality. Hence, it remains uncertain if patients with higher quality of life and self-rated health are more satisfied with staff-related care or if, as we assume, the latter increases the quality of life and self-rated health. Longitudinal studies are required to gain further insights.

## 5. Conclusions

Satisfaction with staff-related care is associated with patient-centered outcomes, such as quality of life and self-rated health. Knowledge about this association is relevant to the physicians’ and nurses’ community as well as to stakeholders. It seems to be a modifiable factor, which is associated with improved self-rated health of patients.

Future research needs to confirm the causality of our findings in longitudinal studies. Afterwards, staff-related care has to be improved, and corresponding interventions need to be designed and implemented.

## Figures and Tables

**Table 1:** Overview of the characteristics of the included patients.

**Variable**	**N = 4925**
	**Mean (SD)/Median [Inter Quartile Range]** **/n (%)**
**Quality of Life (1 low—5 high)**	3.21 (0.90)
**Self-rated health (1 low—5 high)**	3.01 (0.87)
**Satisfaction with physician-related care (0 low—9 high)**	8.19 (1.34) 9.00 [8, 9]
**Satisfaction with nursing-related care (0 low—9 high)**	8.48 (1.13) 9.00 [8, 9]
**Age Groups**	
Up to 20	105 (2.13)
21 to 40	885 (17.70)
41 to 60	1790 (36.35)
60 to 80	1920 (38.98)
80+	225 (4.57)
**Gender**	
Female	1932 (39.23)
Male	2981 (60.53)
Diverse	12 (0.24)
**Mother tongue**	
German	4592 (93.24)
Other	333 (6.76)
**Wards**	
Eyes, otorhinolaryngology, and maxillofacial or oral surgery	1171 (23.98)
Internal medicine	291 (5.91)
Gynaecology and obstetrics	209 (4.24)
Urology including urological oncology	867 (17.60)
Oncology	470 (9.54)
Cardiology, heart, and vascular surgery	682 (13.85)
Neurology and neurosurgery	552 (11.21)
General and trauma surgery	355 (7.21)
Dermatology	80 (1.62)
Others	248 (5.04)

**Table 2:** Multiple linear regression with adjustments for age, gender, mother tongue, and treating ward.

	Unstandardized Beta Coefficient Are Reported (Robust Standard Errors in Parentheses)	*p*-Values
**Model 1—for Quality of life as outcome**		
Intercept	2.32 (0.12)	<0.001
Satisfaction with physician-related care	0.16 (0.01)	<0.001
R-squared	0.09	
**Model 2—for Self-rated Health as outcome**		
Intercept	1.87 (0.12)	<0.001
Satisfaction with physician-related care	0.16 (0.01)	<0.001
R-squared	0.11	
**Model 3—for Quality of life as outcome**		
Intercept	2.48 (0.14)	<0.001
Satisfaction with nurse-related care	0.13 (0.01)	<0.001
R-squared	0.09	
**Model 4—for Self-rated Health as outcome**		
Intercept	2.06 (0.14)	<0.001
Satisfaction with nurse-related care	0.14 (0.01)	<0.001
R-squared	0.07	
**Observations in all models**	4925	

## Data Availability

All data was obtained from the UKE quality management department after request.

## Supplementary Materials

The following supporting information can be downloaded at: https://www.mdpi.com/article/10.3390/healthcare11050775/s1, Table S1: Multiple linear regression on satisfaction with physician-related care and quality of life with adjustments for age, gender, mother tongue, and treating ward.; Table S2: Multiple linear regression on satisfaction with physician-related care and self-rated health with adjustments for age, gender, mother tongue, and treating ward.; Table S3: Multiple linear regression on satisfaction with nurse-related care and quality of life with adjustments for age, gender, mother tongue, and treating ward.; Table S4: Multiple linear regression on satisfaction with nurse-related and care self-rated health with adjustments for age, gender, mother tongue, and treating ward. Figure S1: Directed acyclic graph to identify underling confounding variables in the associations between inpatients satisfaction with care and quality of life as well as self-rated health.
