# Supplementary material for: Patients Satisfied with Care Report Better Quality of Life and Self-Rated Health—Cross-Sectional Findings Based on Hospital Quality Data
Source: Healthcare (Basel). 2023 Mar 6;11(5):775. doi: 10.3390/healthcare11050775 (PMC10001220; doi:10.3390/healthcare11050775)
Supplement: Supplementary file 1 [file healthcare-11-00775-s001.zip › healthcare-2208132-supplementary-materials.pdf]

Supplementary

Figure S1 Directed acyclic graph to identify underling confounding variables in the associations between inpatients satisfaction with care and quality of life as well as self-rated health

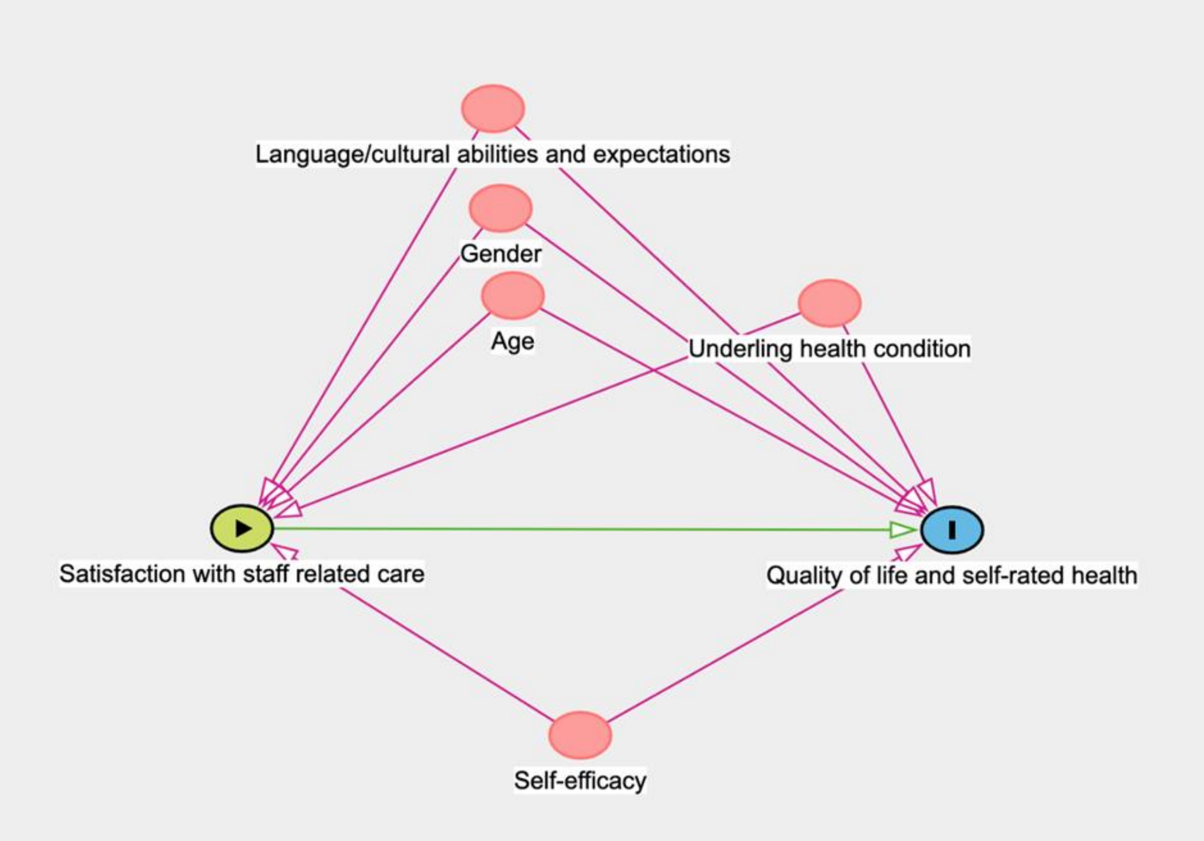

Table S1 Multiple linear regression on satisfaction with physician-related care and quality of life with adjustments for age, gender, mother tongue, and treating ward.

	Estimate	Robust Standard Error	P-value
**Intercept**	2.32	0.12	<0.001
**Satisfaction with physician-related care**	0.16	0.01	<0.001
**Treating ward (ref. eyes. otorhinolaryngology and maxillofacial or oral surgery)**			
internal medicine	-0.20	0.06	<0.001
gynecology and obstetrics	0.12	0.06	0.081
urology including urological oncology	-0.06	0.04	0.174
oncology	-0.25	0.05	<0.001
cardiology, heart, and vascular surgery	-0.07	0.04	0.085
neurology und neurosurgery	-0.25	0.05	<0.001
general and trauma surgery	-0.18	0.05	<0.001
dermatology	-0.27	0.11	0.007
others	-0.28	0.06	<0.001
**Age group (ref. <20 years)**			
21 - 40 years	-0.11	0.09	0.230
41 - 60 years	-0.33	0.08	<0.001

61 - 80 years	-0.44	0.08	<0.001
> 80 years	-0.69	0.10	<0.001
**Gender (ref. female)**			
male	0.08	0.03	0.003
diverse	0.29	0.27	0.245
**Mother tongue (ref. German)**			
Other mother tongue	0.08	0.05	0.121

Table S2 Multiple linear regression on satisfaction with physician-related care and self-rated health with adjustments for age, gender, mother tongue, and treating ward.

	**Estimate**	**Robust Standard Error**	**P-value**
**Intercept**	1.87	0.12	<0.001
**Satisfaction with physician-related care**	0.16	0.01	<0.001
**Treating ward (ref. eyes. otorhinolaryngology and maxillofacial or oral surgery)**			
internal medicine	-0.24	0.06	<0.001
gynecology and obstetrics	0.06	0.06	0.332
urology including urological oncology	0.06	0.04	0.134
oncology	-0.17	0.05	<0.001
cardiology, heart, and vascular surgery	-0.02	0.04	0.612
neurology und neurosurgery	-0.19	0.04	<0.001
general and trauma surgery	-0.14	0.05	0.003
dermatology	-0.34	0.09	<0.001
others	-0.16	0.06	0.007
**Age group (ref. <20 years)**			
21 - 40 years	0.03	0.09	0.684
41 - 60 years	-0.22	0.09	0.008
61 - 80 years	-0.31	0.09	<0.001
> 80 years	-0.50	0.11	<0.001
**Gender (ref. female)**			
male	0.11	0.03	<0.001
diverse	0.22	0.24	0.363
**Mother tongue (ref. German)**			
Other mother tongue	0.17	0.05	<0.001

Table S3 Multiple linear regression on satisfaction with nurse-related care and quality of life with adjustments for age, gender, mother tongue, and treating ward.

	**Estimate**	**Robust Standard Error**	**P-value**
**Intercept**	2.48	0.14	<0.001
**Satisfaction with nurse-related care**	0.13	0.01	<0.001
**Treating ward (ref. eyes. otorhinolaryngology and maxillofacial or oral surgery)**			
internal medicine	-0.21	0.06	<0.001
gynecology and obstetrics	0.12	0.07	0.069
urology including urological oncology	-0.04	0.04	0.304
oncology	-0.26	0.05	<0.001
cardiology, heart, and vascular surgery	-0.09	0.04	0.037
neurology und neurosurgery	-0.27	0.05	<0.001
general and trauma surgery	-0.20	0.05	<0.001
dermatology	-0.28	0.11	0.005
others	-0.31	0.07	<0.001
**Age group (ref. <20 years)**			
21 - 40 years	-0.11	0.09	0.228
41 - 60 years	-0.33	0.09	<0.001
61 - 80 years	-0.41	0.09	<0.001
> 80 years	-0.68	0.10	<0.001
**Gender (ref. female)**			
male	0.08	0.03	0.003
diverse	0.16	0.28	0.524
**Mother tongue (ref. German)**			
Other mother tongue	0.09	0.05	0.073

Table S4 Multiple linear regression on satisfaction with nurse-related and care self-rated health with adjustments for age, gender, mother tongue, and treating ward.

	**Estimate**	**Robust Standard Error**	**P-value**
**Intercept**	2.06	0.14	<0.001
**Satisfaction with nurse-related care**	0.14	0.01	<0.001
**Treating ward (ref. eyes. otorhinolaryngology and maxillofacial or oral surgery)**			
internal medicine	-0.25	0.05	<0.001
gynecology and obstetrics	0.07	0.07	0.287
urology including urological oncology	0.07	0.04	0.067
oncology	-0.18	0.05	<0.001
cardiology, heart, and vascular surgery	-0.04	0.04	0.355
neurology und neurosurgery	-0.22	0.04	<0.001
general and trauma surgery	-0.16	0.05	0.001
dermatology	-0.36	0.09	<0.001
others	-0.19	0.06	0.002
**Age group (ref. <20 years)**			
21 - 40 years	0.03	0.10	0.717
41 - 60 years	-0.21	0.09	0.012
61 - 80 years	-0.28	0.09	<0.001
> 80 years	-0.48	0.11	<0.001
**Gender (ref. female)**			
male	0.11	0.03	<0.001
diverse	0.08	0.26	0.751
**Mother tongue (ref. German)**			
Other mother tongue	0.19	0.05	<0.001
